# Supplementary material for: Dramatic Suppression of Lipogenesis and No Increase in Beta-Oxidation Gene Expression Are among the Key Effects of Bergamot Flavonoids in Fatty Liver Disease
Source: Antioxidants (Basel). 2024 Jun 25;13(7):766. doi: 10.3390/antiox13070766 (PMC11273501; doi:10.3390/antiox13070766)

Supplementary Material

Dramatic suppression of lipogenesis and no increase in beta-oxidation are among the main gene expression effects of bergamot flavonoids in fatty liver disease.

Maddalena Parafati[1*], Daniele La Russa[2*], Antonella Lascala[1*], Francesco Crupi[1], Concetta Riillo[1], Bartosz Fotschki[3], Vincenzo Mollace[1] and Elzbieta Janda[1#]

[1]Department of Health Sciences, Magna Graecia University, Campus Germaneto, 88100 Catanzaro, Italy
[2]Department of Biology, Ecology and Earth Sciences, University of Calabria, 87036 Rende, Cosenza, Italy
[3]Department of Biological Function of Food, Institute of Animal Reproduction and Food Research, Polish Academy of Sciences, Olsztyn, Poland

* these authors contributed equally to this work

# Correspondence: **janda@unicz.it**

Supplementary Material and Methods

***Liver histology and* lipid droplets (LDs) staining and analysis**

10 μm thick frozen sections of the perfused liver from the central portion of the main lobe were prepared as previously described [10]. For lipid droplets (LDs) staining, the liver sections mounted on slides were rinsed with distilled water and immersed in 60% isopropanol (Sigma-Aldrich, St. Louis, MO, USA) and then LDs were stained with freshly prepared Oil Red O (ORO, Sigma-Aldrich) working solution for 15 min. The sections were then rinsed with 60% isopropanol and washed with de-ionized water three times for 30 sec. Then, half of the sections were counterstained with Mayer's hematoxylin (05-06002/L; Bio-Optica Milano Spa, Milano, Italy) for 60 sec to visualize the nuclei, rinsed with running tap water for 10 min and then covered with a film of embedding medium *SlowFade Gold* (Cat. No. S36936; Life Technologies, Thermo Fischer Scientific, Road Grand Island, NY, USA) and a coverslip. Equivalent ORO and ORO plus hematoxylin-stained liver sections of each experimental group were examined in bright-field with Leica Microscope DM4000B (Leica Microsystems GmbH, Wetzlar, Germany) equipped with 10×, 40×, and 100× objective lenses. For quantitative LDs accumulation analysis, digital images of ORO-stained sections were acquired by Sp2 confocal microscopy (Leica Microsystems GmbH) by using a TRITC wide emission filter (540–580 nm) and accumulation settings. For each liver section, at least five independent images from equivalent central lobe areas were captured at 40× magnification. Within each area, four different regions were chosen randomly and acquired with zoom at 4.65. These images were processed and analyzed using a semiautomatic procedure implemented in Image J2x software.

Figure S1

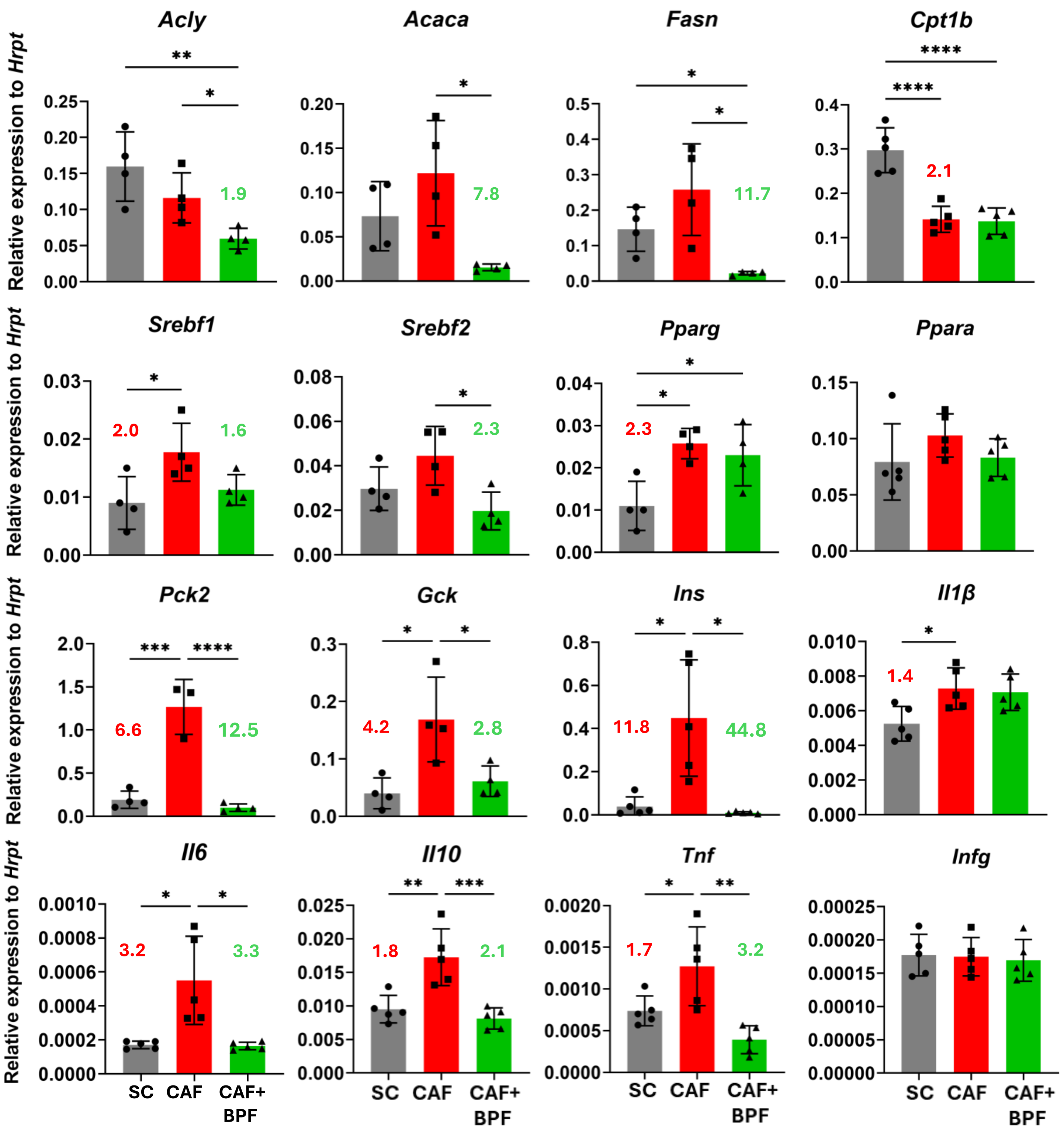

Figure S1. The expression level of selected genes was analyzed by RT-qPCR with an independent set of primers in each animal separately. The bar represents the mean ± SD of expression data from n = 4 to 5 rat livers relative to the house-keeping control (*Hrpt1*). Statistical analysis: One-way ANOVA followed by Tukey's post-test or uncorrected Fisher's LSD test, except for ***Acaca, Fasn, Ins and Il6*** in which Brown-Forsythe test followed by unpaired t test with Welch's correction was applied. * $p \leq 0.05$, ** $p \leq 0.01$, *** $p \leq 0.001$, ****$p \leq 0.0001$. Red numbers indicate fold change in CAF compared to SC group. Green numbers indicate fold change in CAF+BPF group compared to CAF group.

ORIGINAL BLOTS

FIGURE 2° Panel 1

OVEREXPOSURE

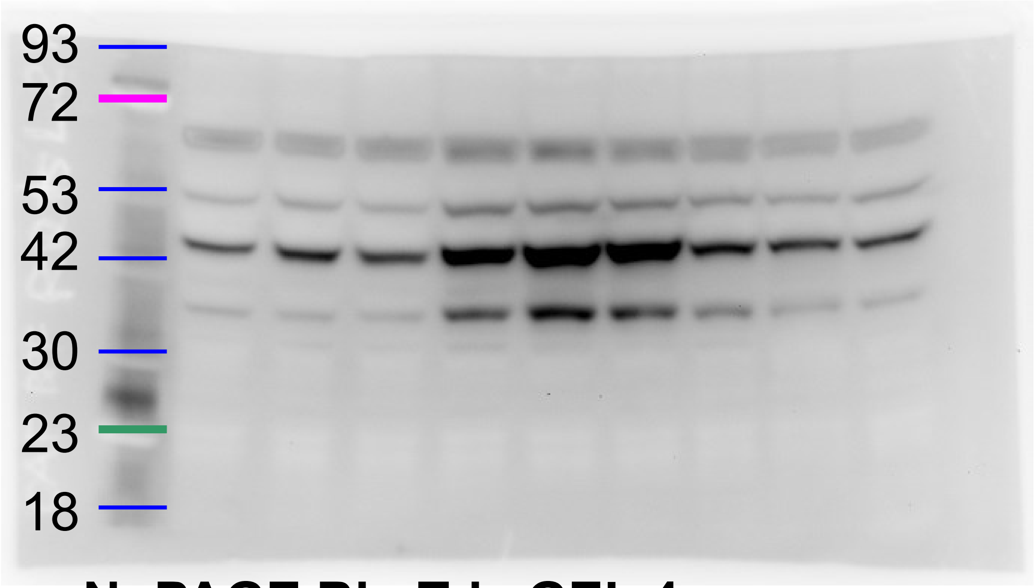

NuPAGE Bis-Tris GEL 4-12%

EXPOSURE: 60s

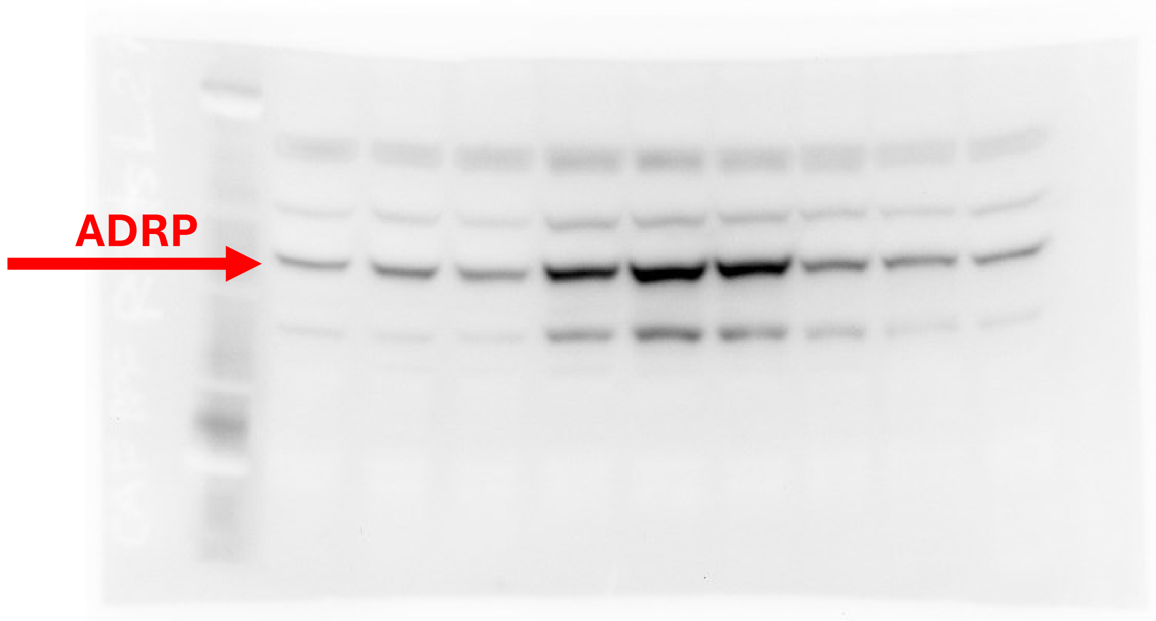

ADRP
FIGURE 2 E and
FIGURE 8 A   PANEL 5

Loading control to this blot is the same as in the Figure 8A.
See GAPDH in Figure 8A, Panel 6.

Amid Black

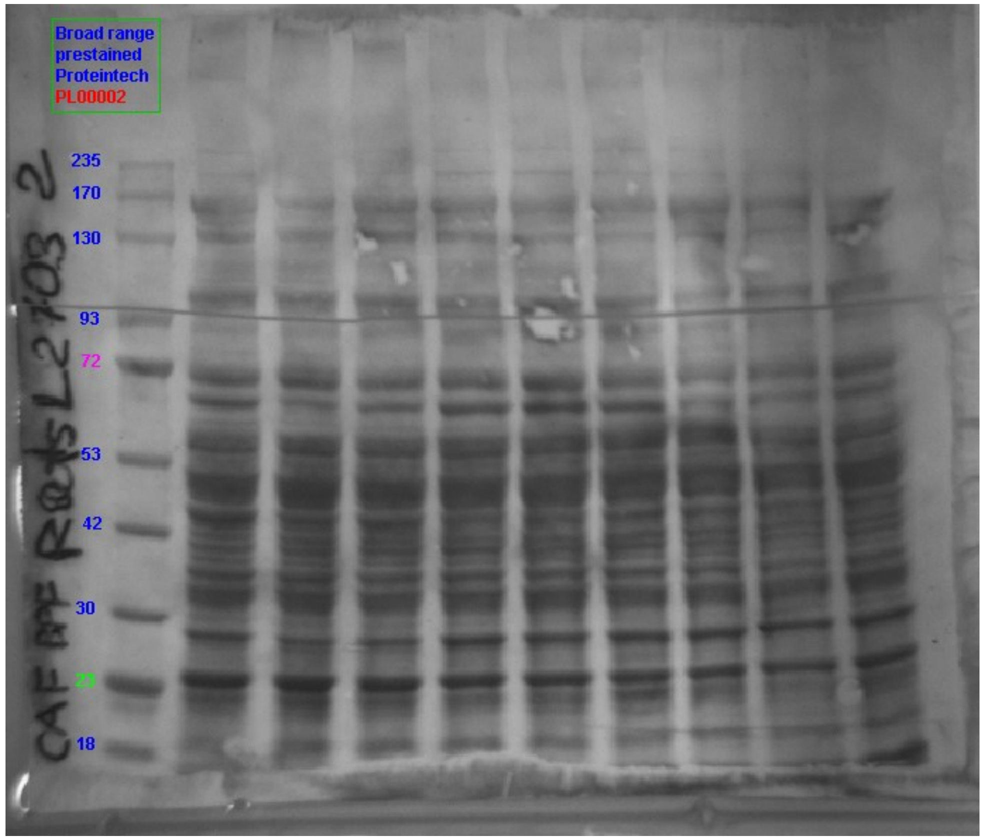

FIGURE 8A Panel 1

EXP: 300s

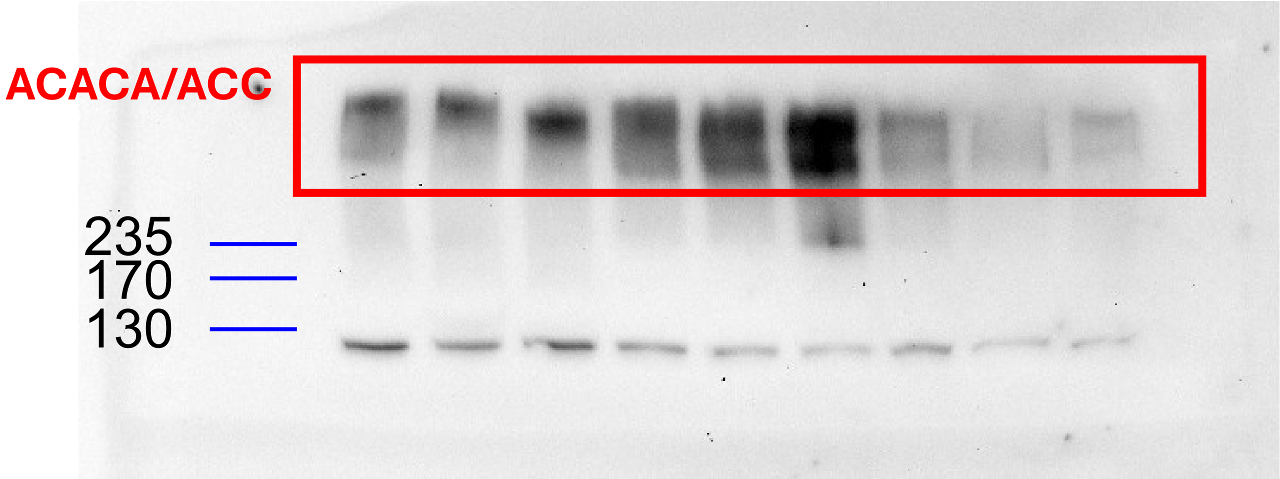

EXP: 60s

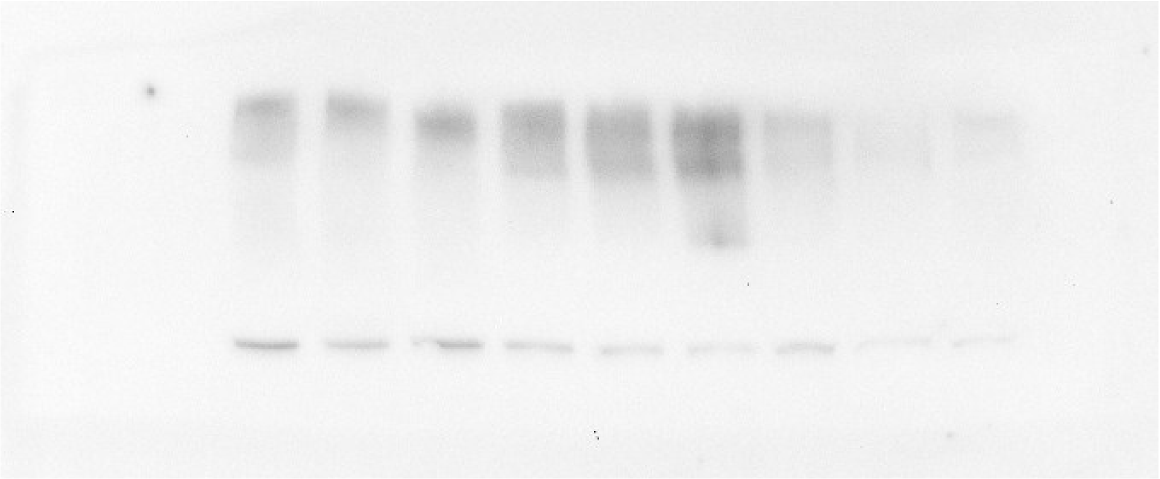

NuPAGE Bis-Tris GEL 4-12%
Upper part

ACACA
FIGURE 8A
PANEL 1

Amid Black

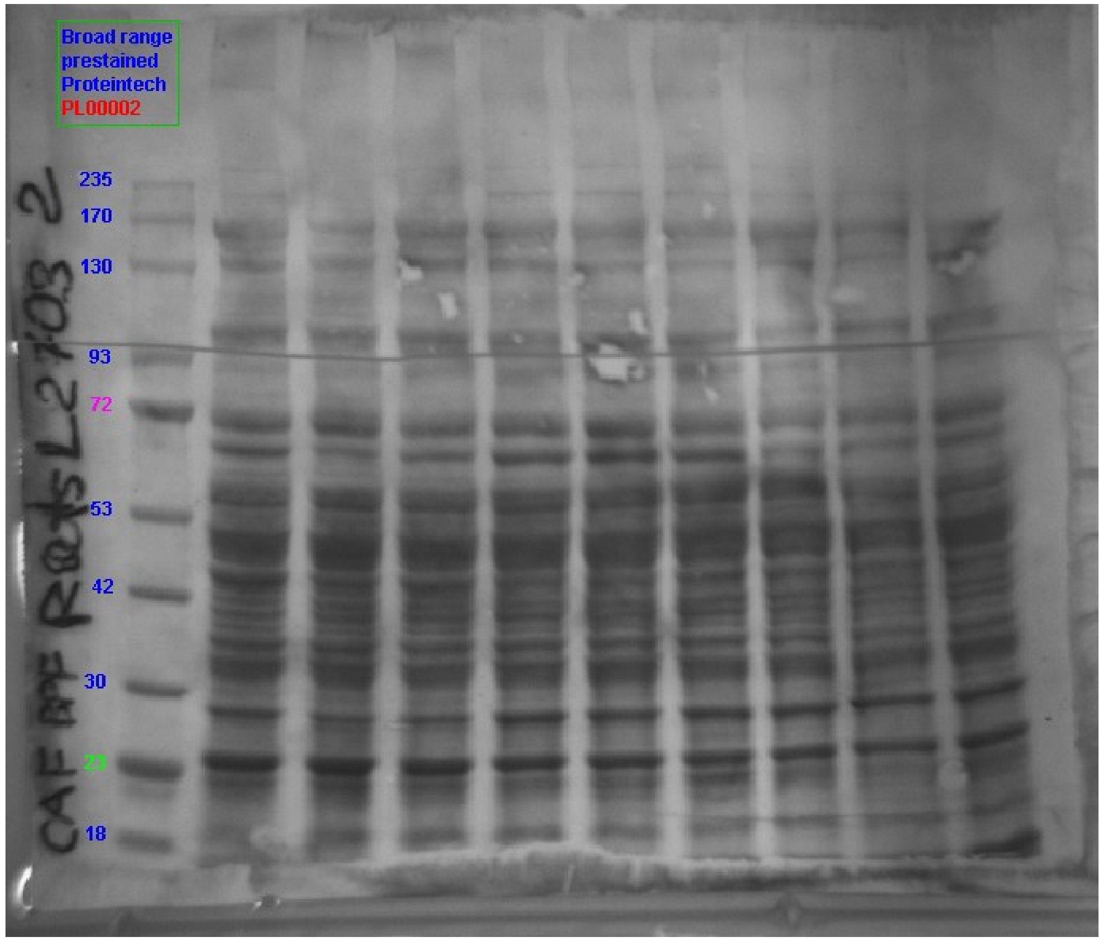

FIGURE 8A Panel 2

OVEREXPOSURE

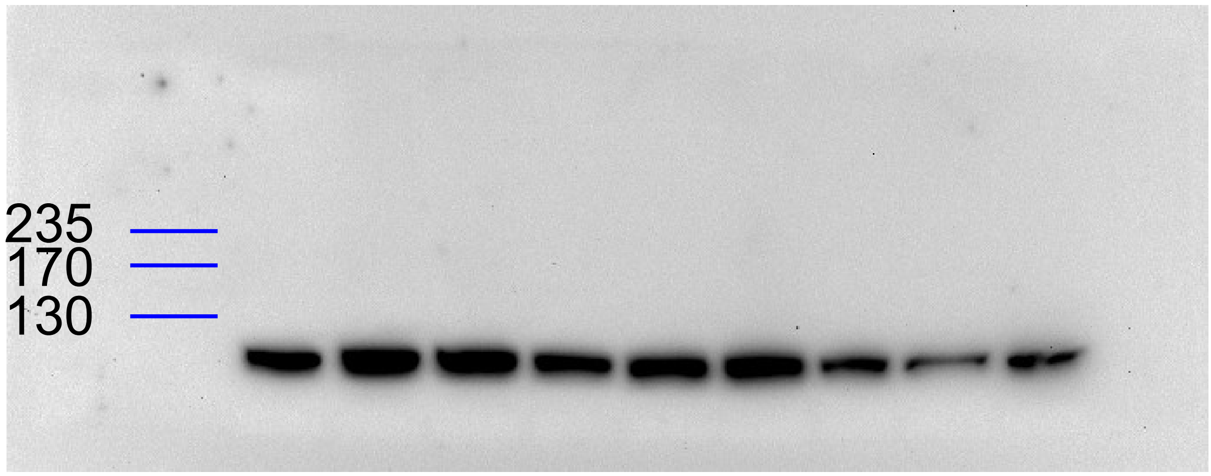

EXPOSURE: 60s

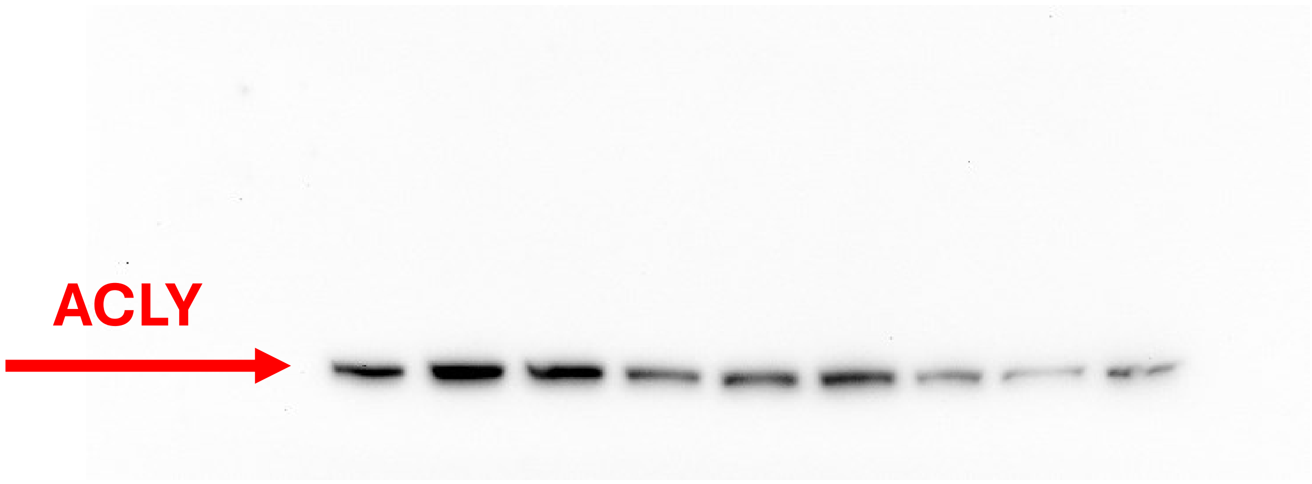

NuPAGE Bis-Tris GEL 4-12%, upper part

ACLY
FIGURE 8°
Panel 2

Amid Black

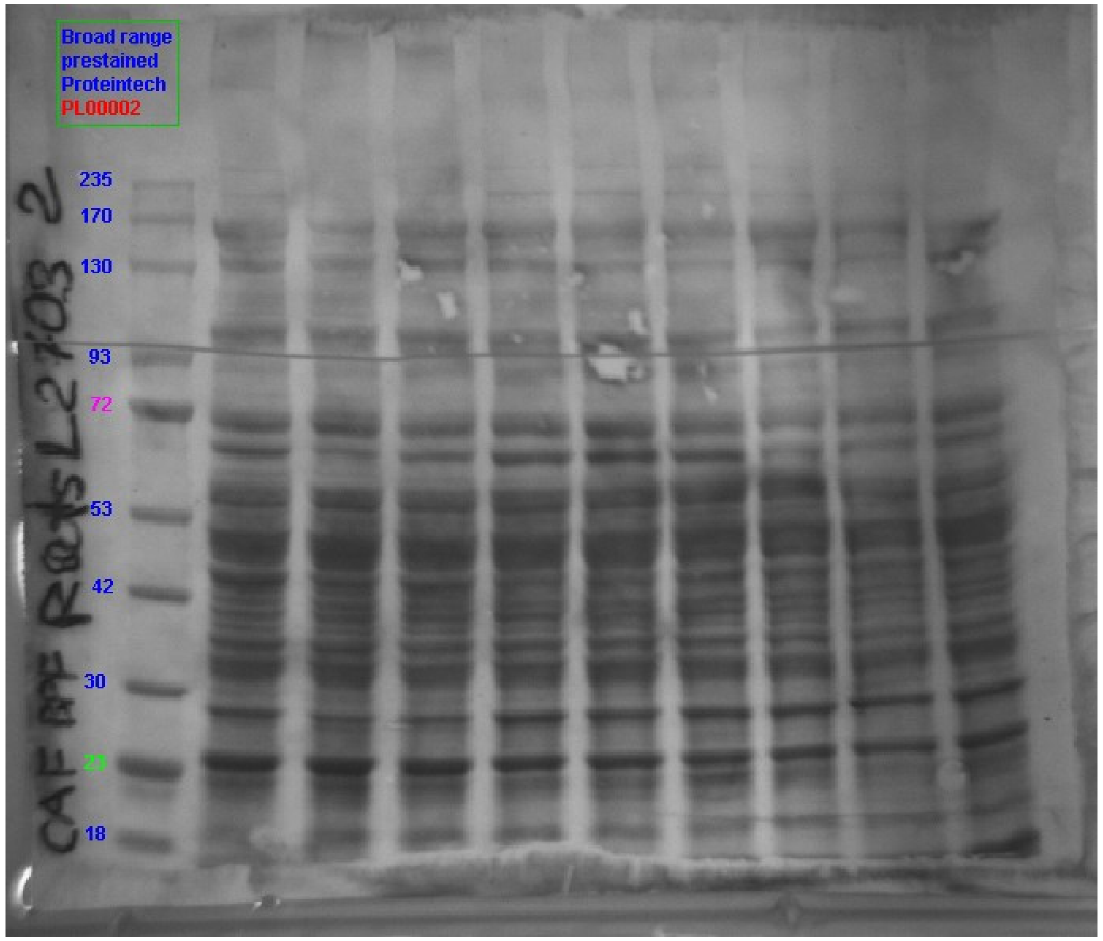

FIGURE 8A Panel 3

OVEREXPOSURE

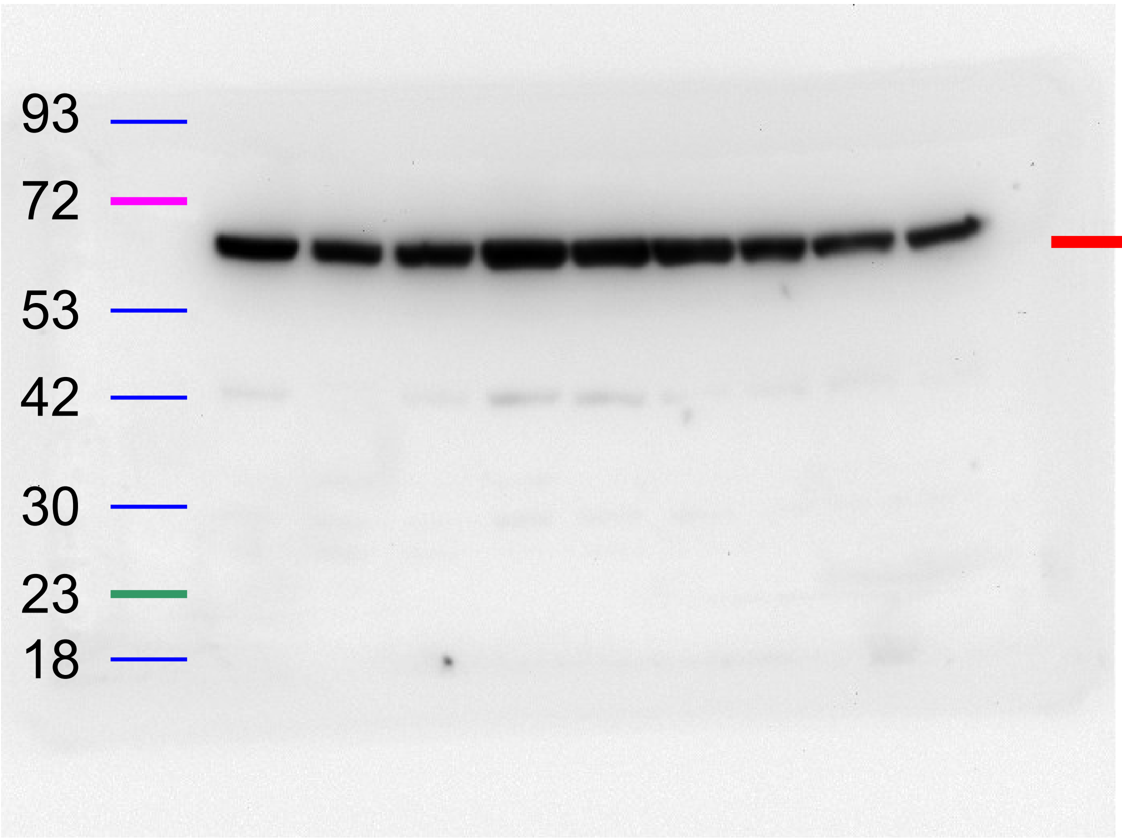

EXPOSURE: 180s

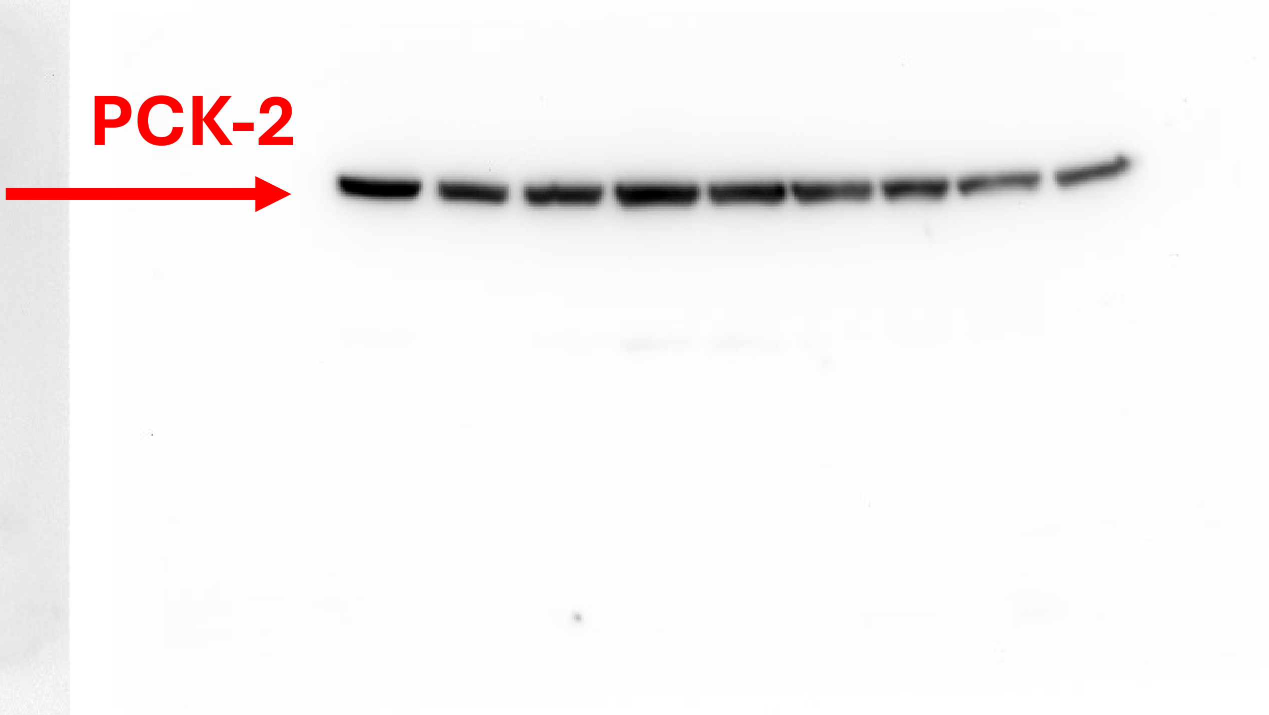

NuPAGE Bis-Tris GEL 4-12%

PCK-2
FIGURE 8A
Panel 3

Amid Black

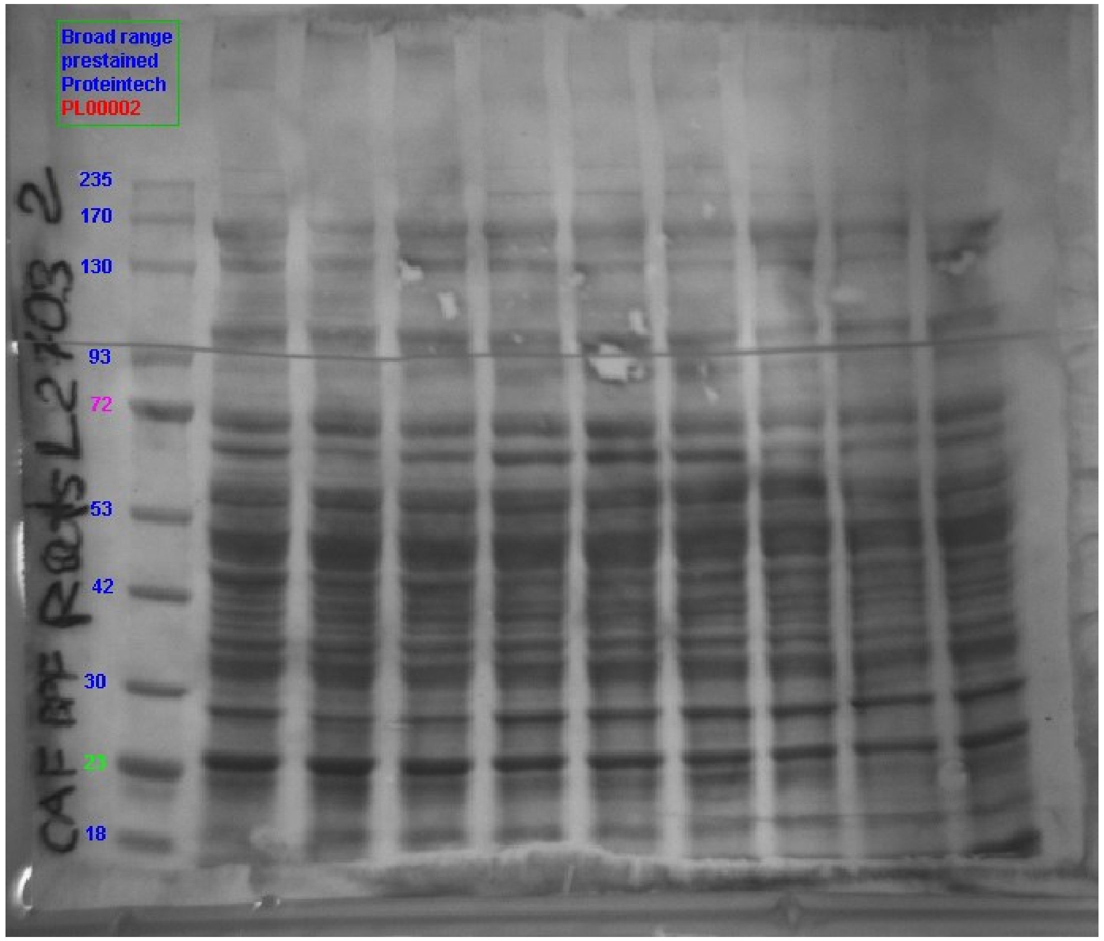

FIGURE 8A Panel 4

EXPOSURE: 300s

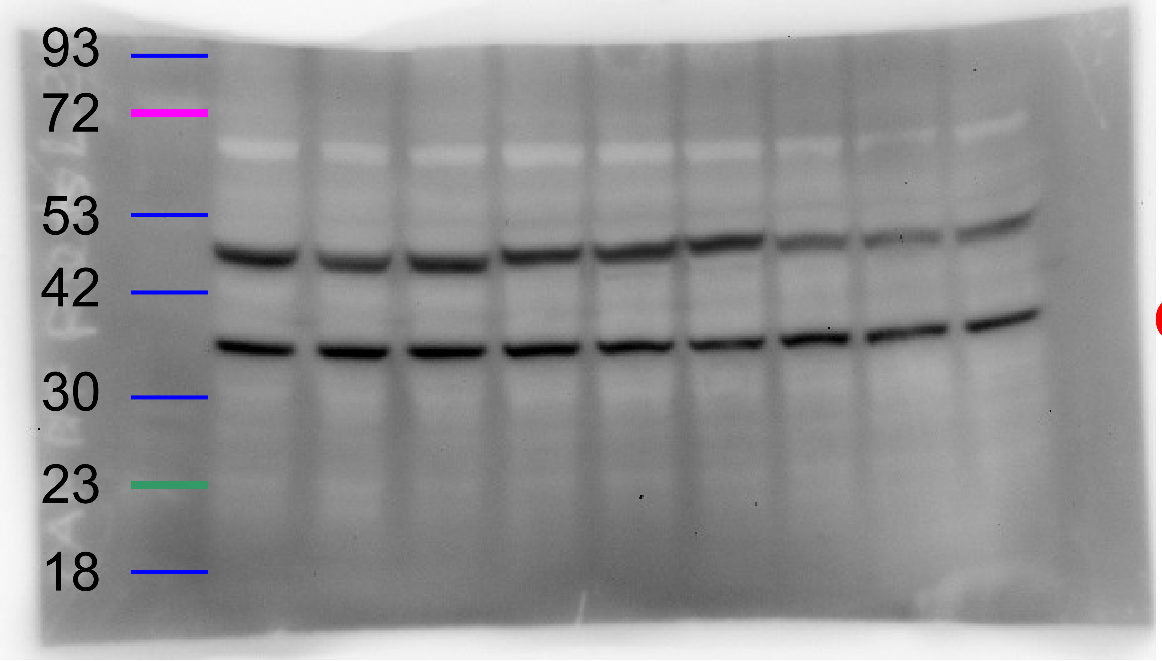

NuPAGE Bis-Tris GEL 4-12%, lower part

EXPOSURE: 60s

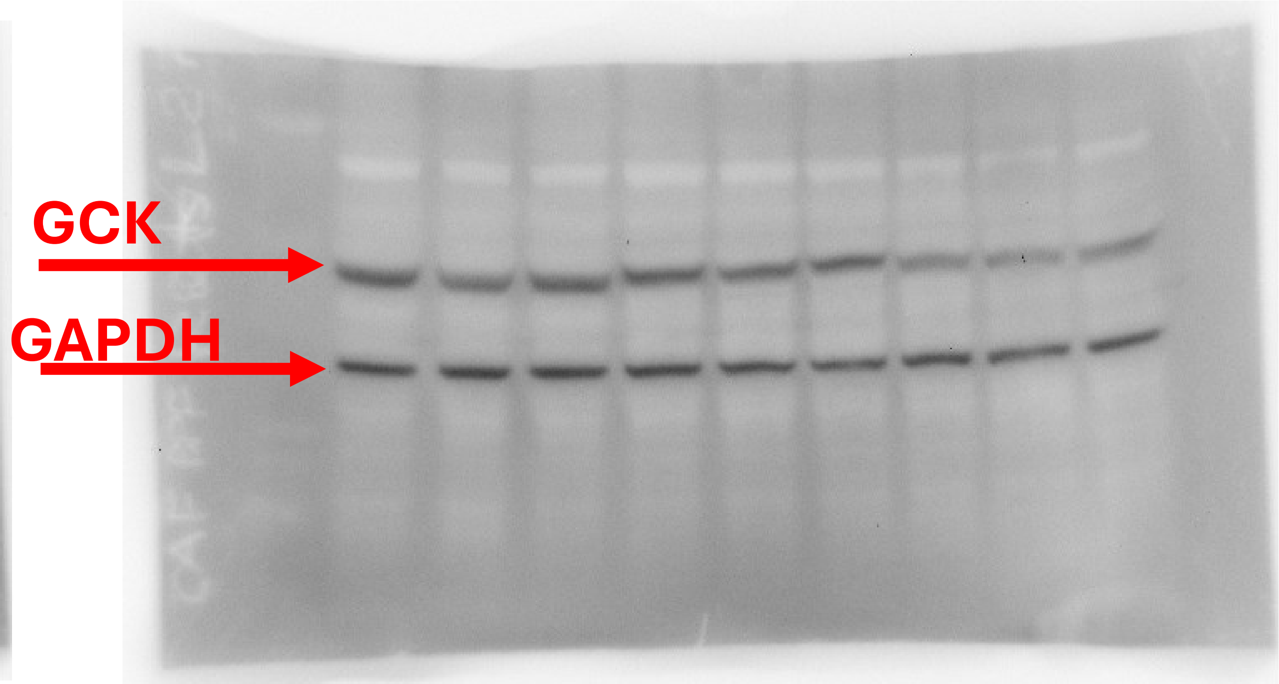

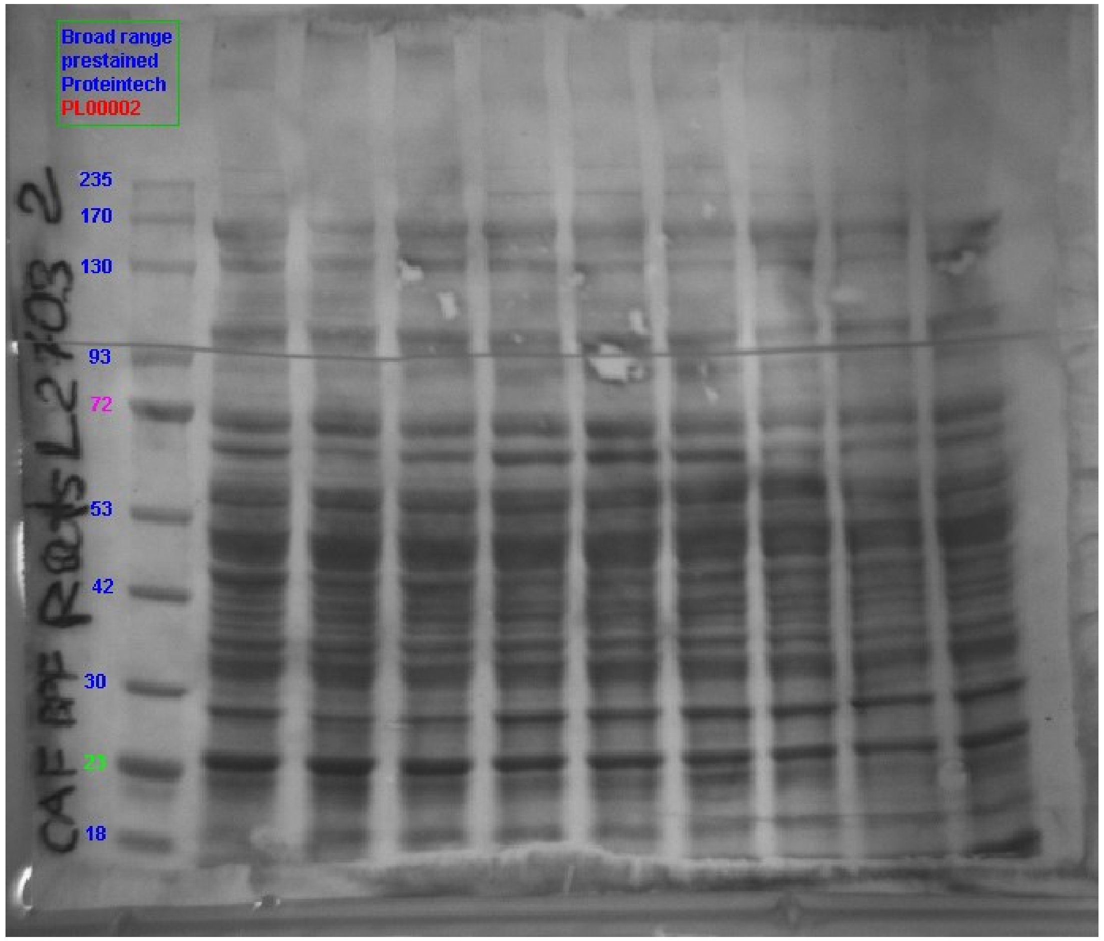

Amid Black

GCK
FIGURE 8°
Panel 4

FIGURE 8A Panel 6

OVEREXPOSURE

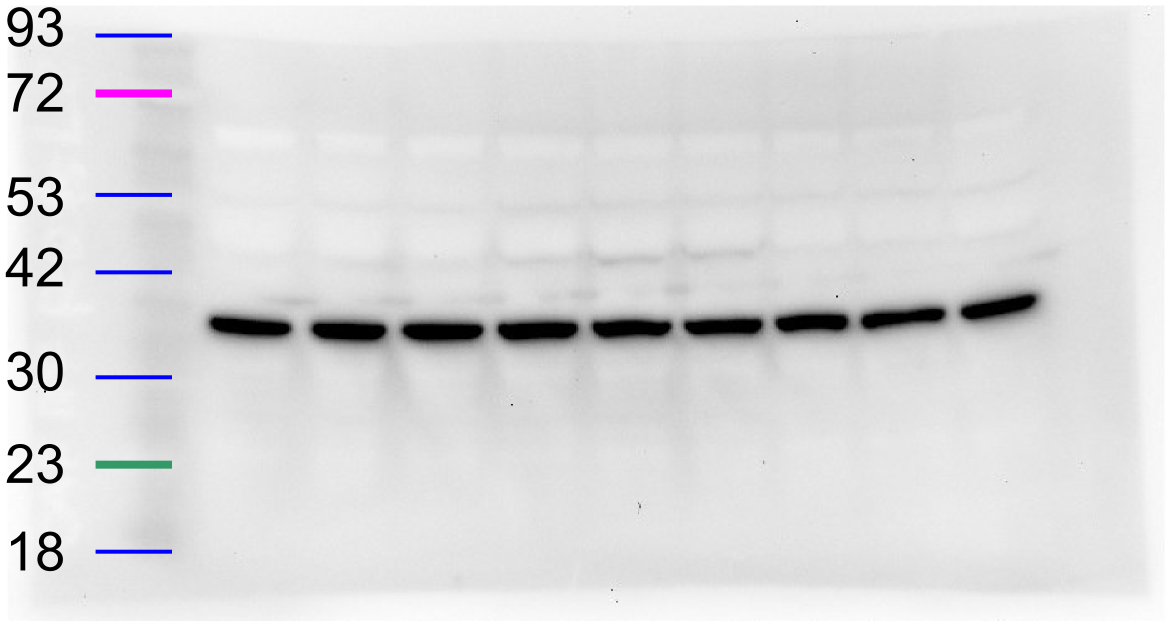

NuPAGE Bis-Tris GEL 4-12%, lower part

EXPOSURE: 60s

GAPDH

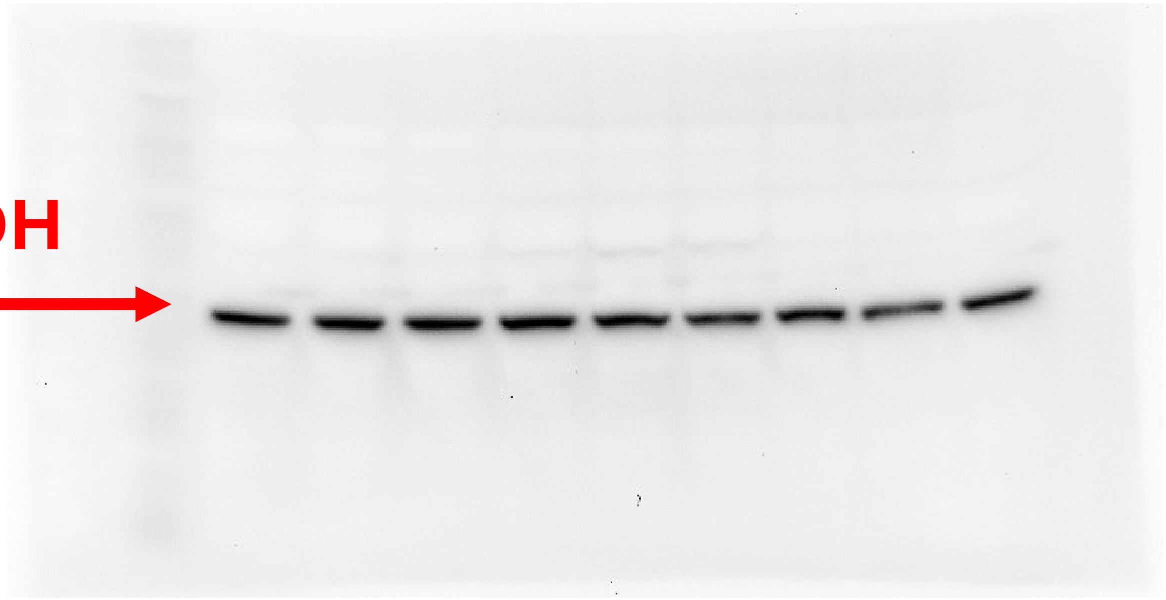

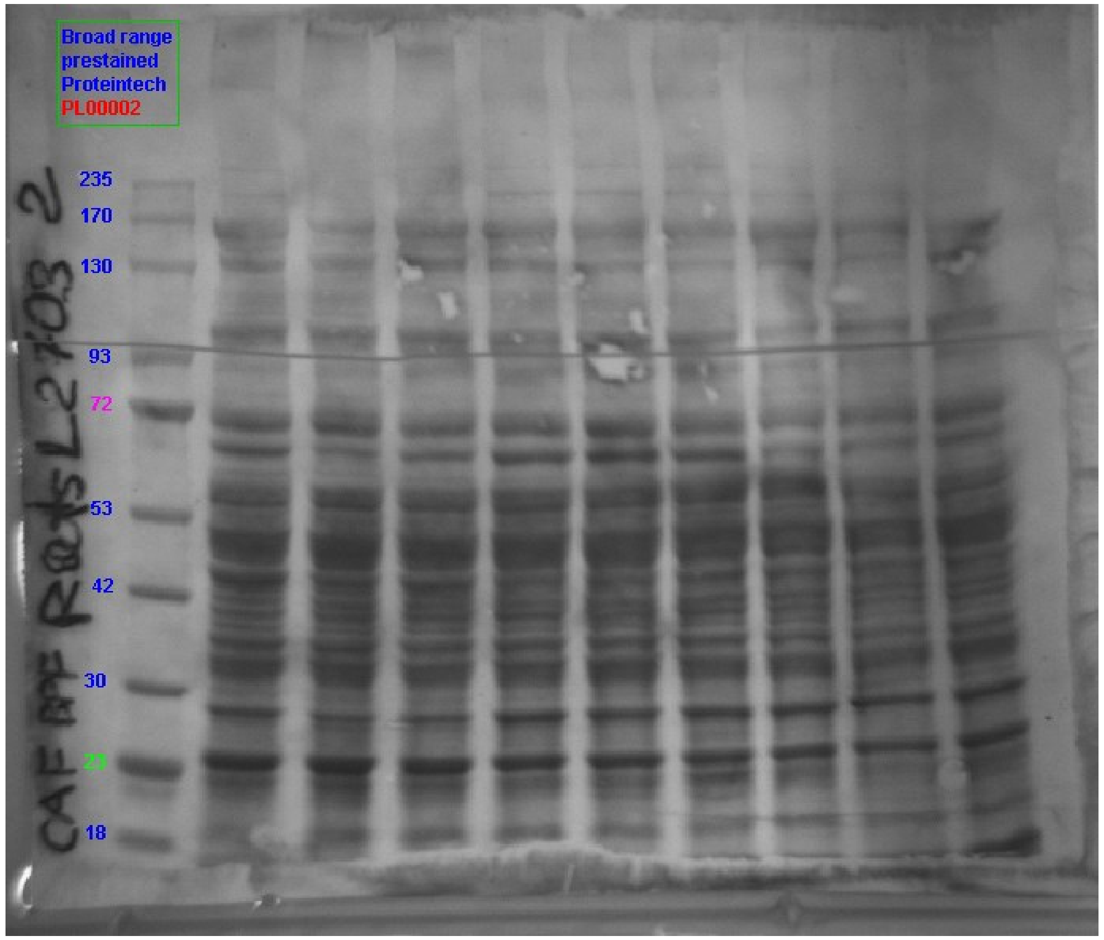

Amid Black

GAPDH
FIGURE 8A
PANEL 6

FIGURE 8C Panel 1

OVEREXPOSURE

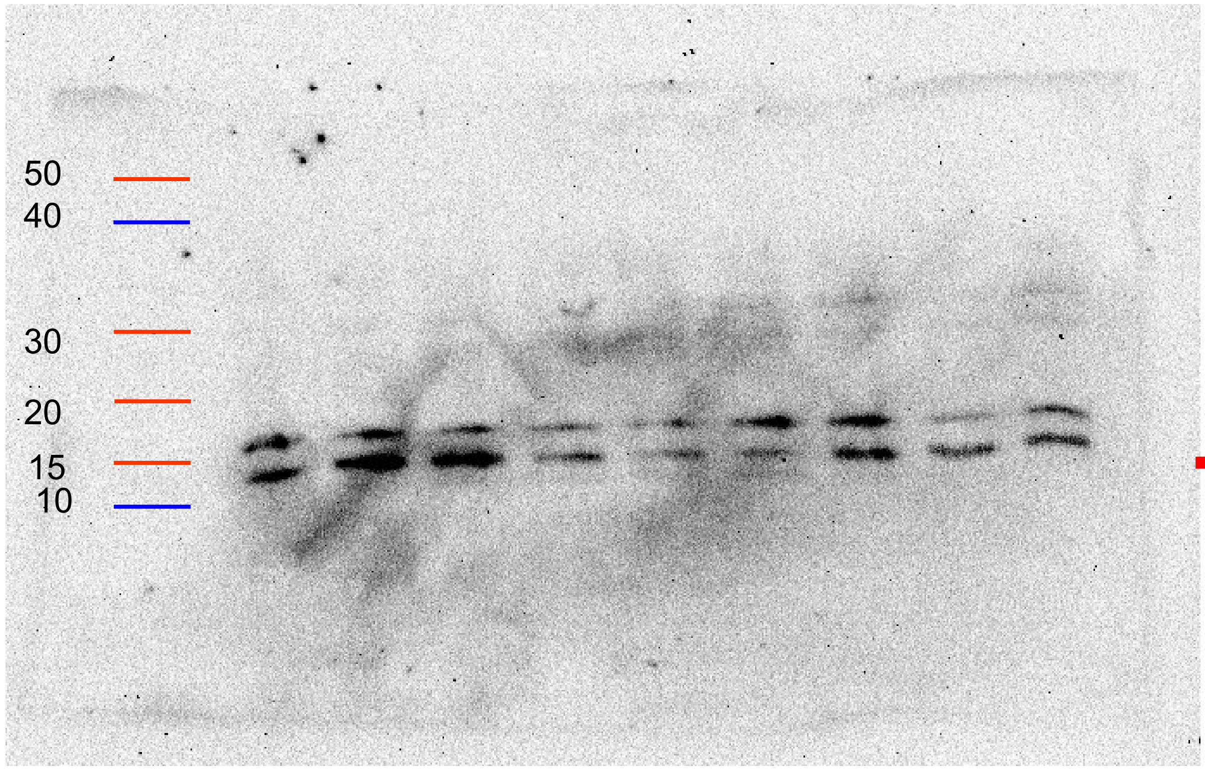

SDS-PAGE GEL 15%

EXPOSURE: 300s

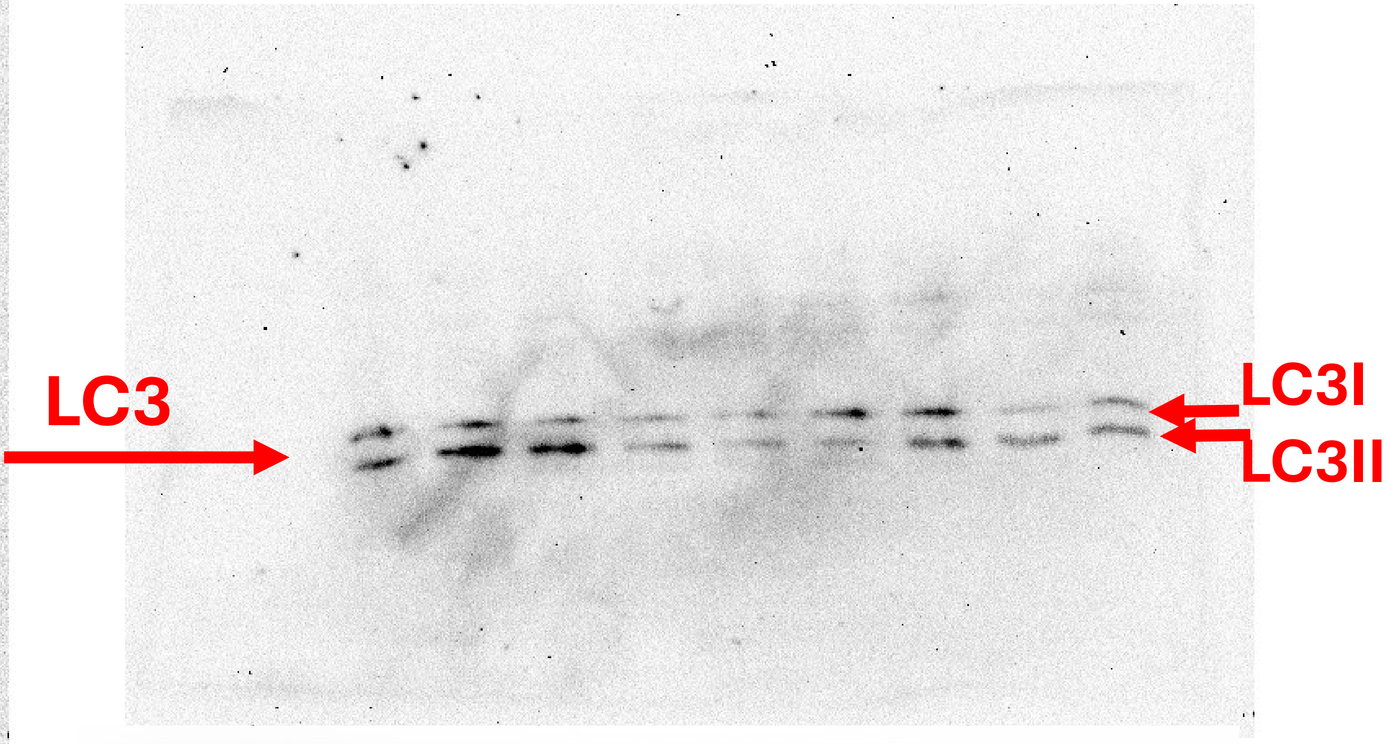

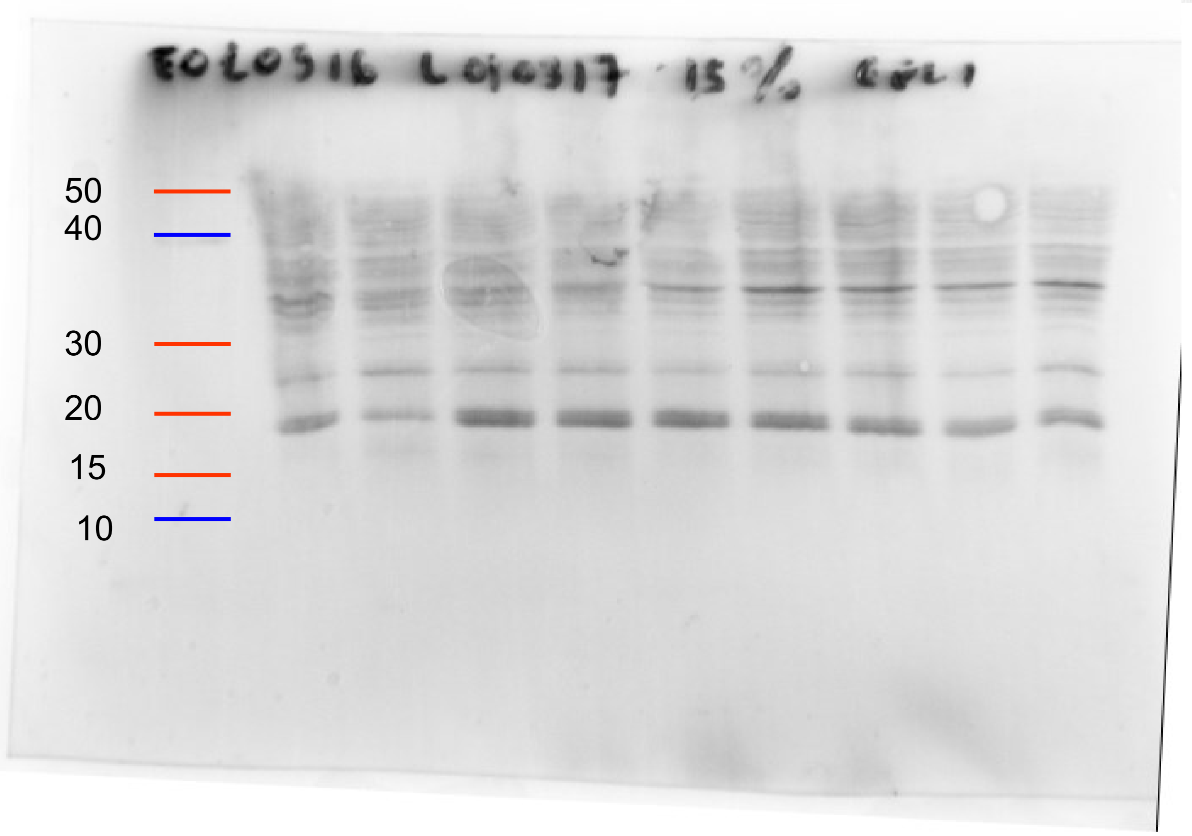

Amid Black

LC3
FIGURE 8C
PANEL 1

FIGURE 8C Panel 2

OVEREXPOSURE

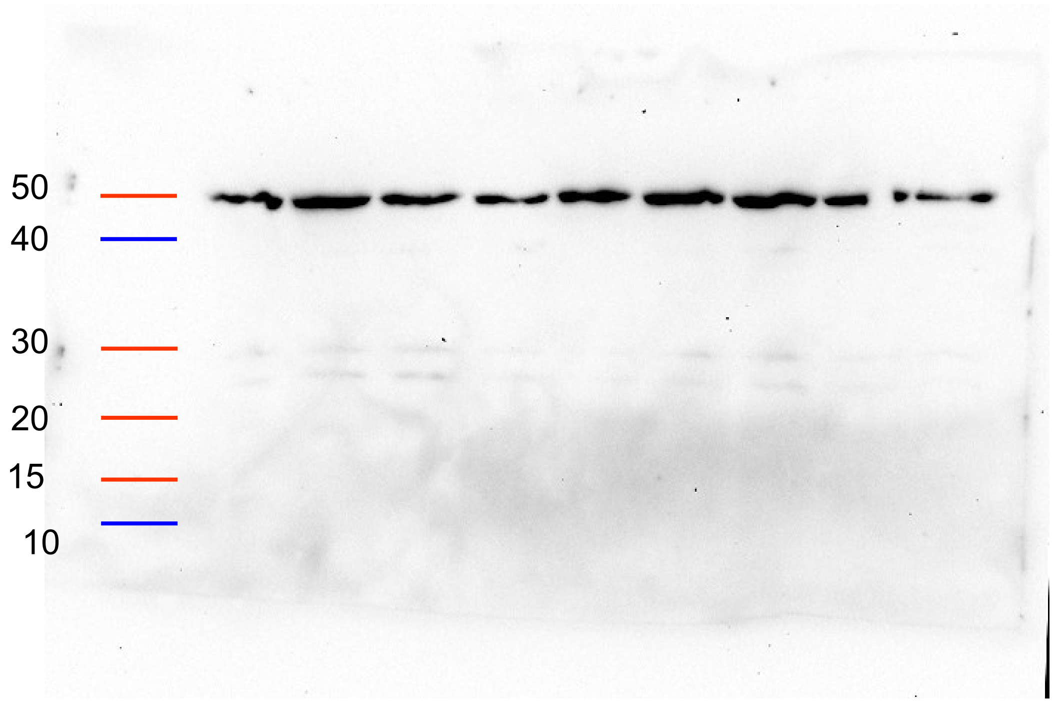

EXPOSURE: 300s

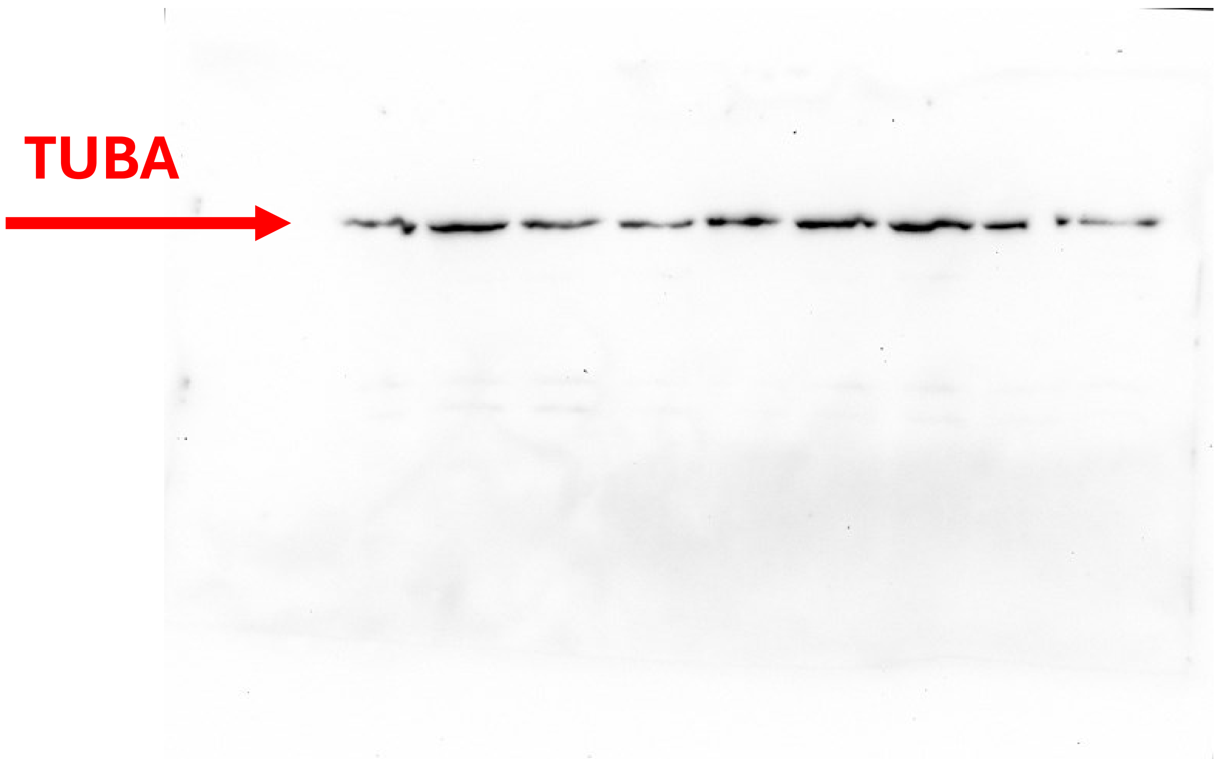

SDS-PAGE GEL 15%

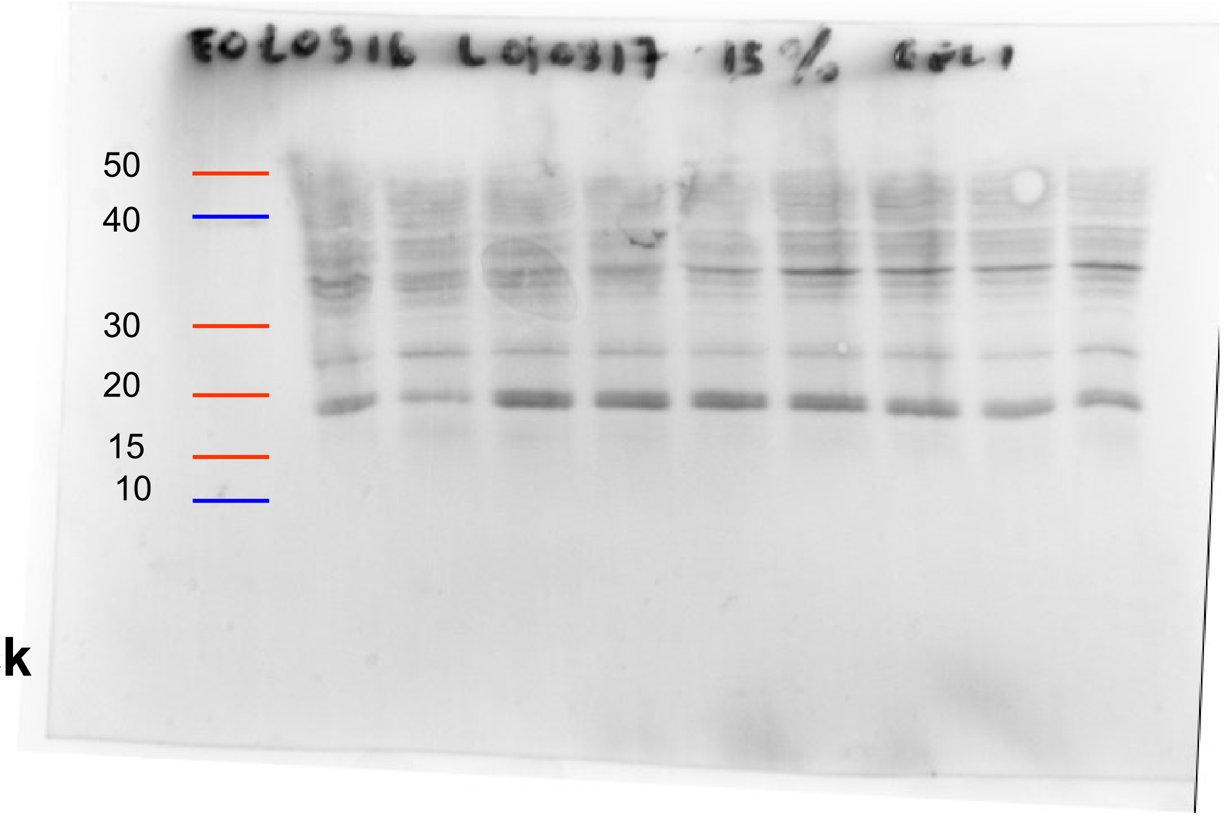

TUBA
FIGURE 8C
PANEL 2

Amid Black

FIGURE 8C Panel 3

OVEREXPOSURE

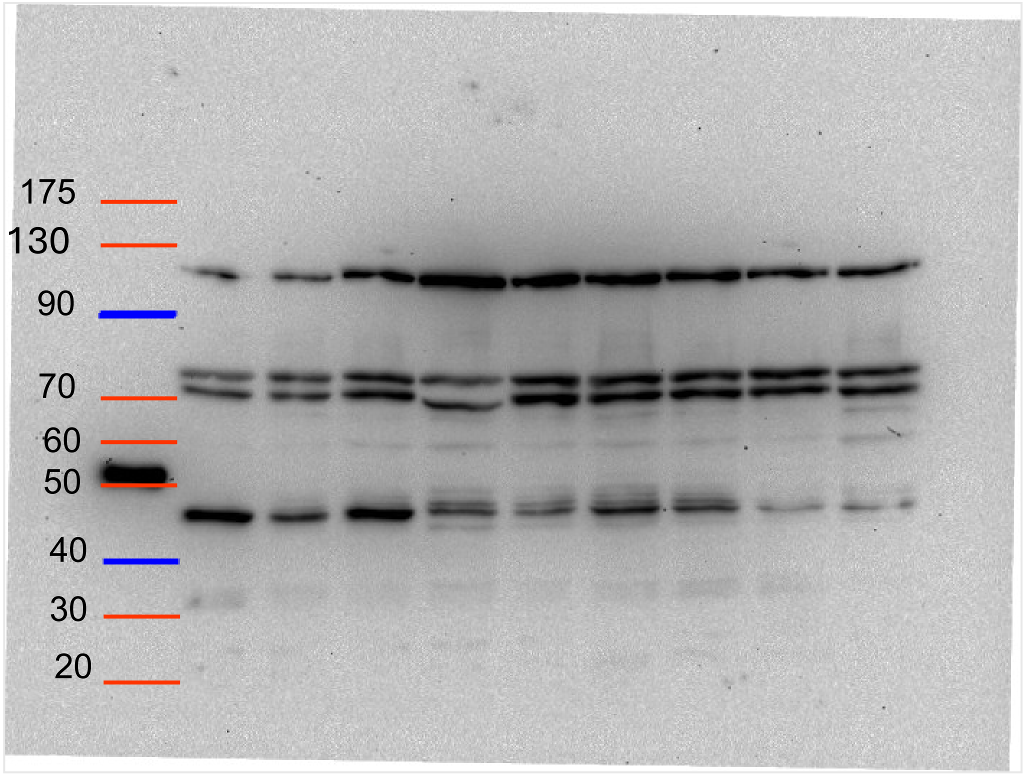

SDS-PAGE GEL 8%

EXPOSURE: 300s

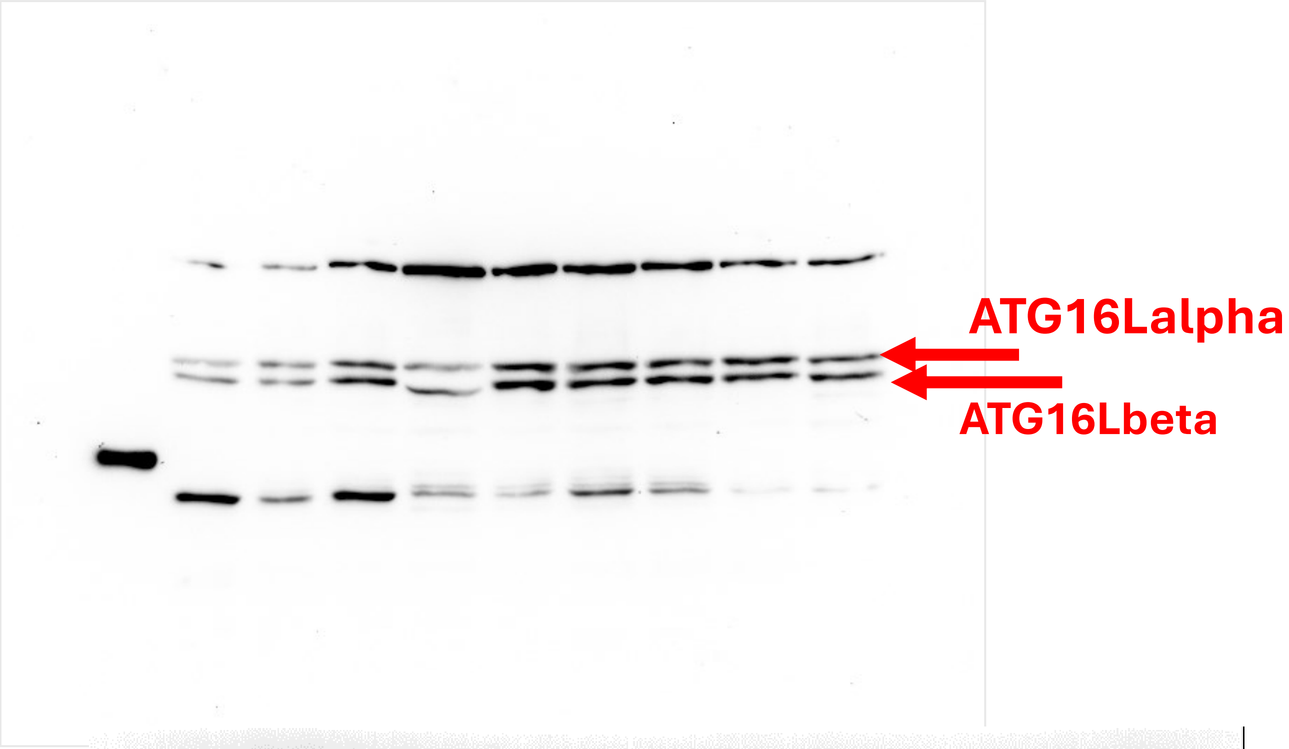

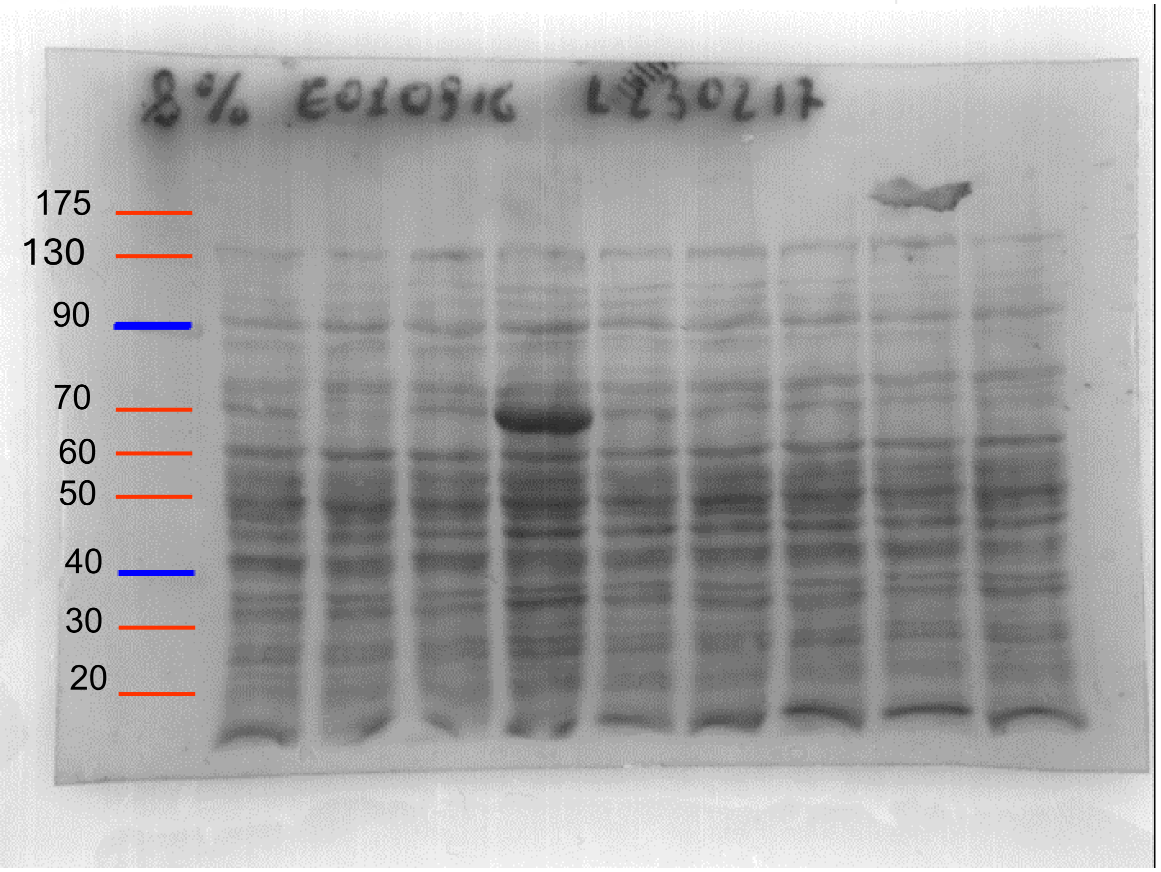

Amid Black

ATG16
FIGURE 8C
PANEL 3

FIGURE 8C Panel 4

EXPOSURE: 300s

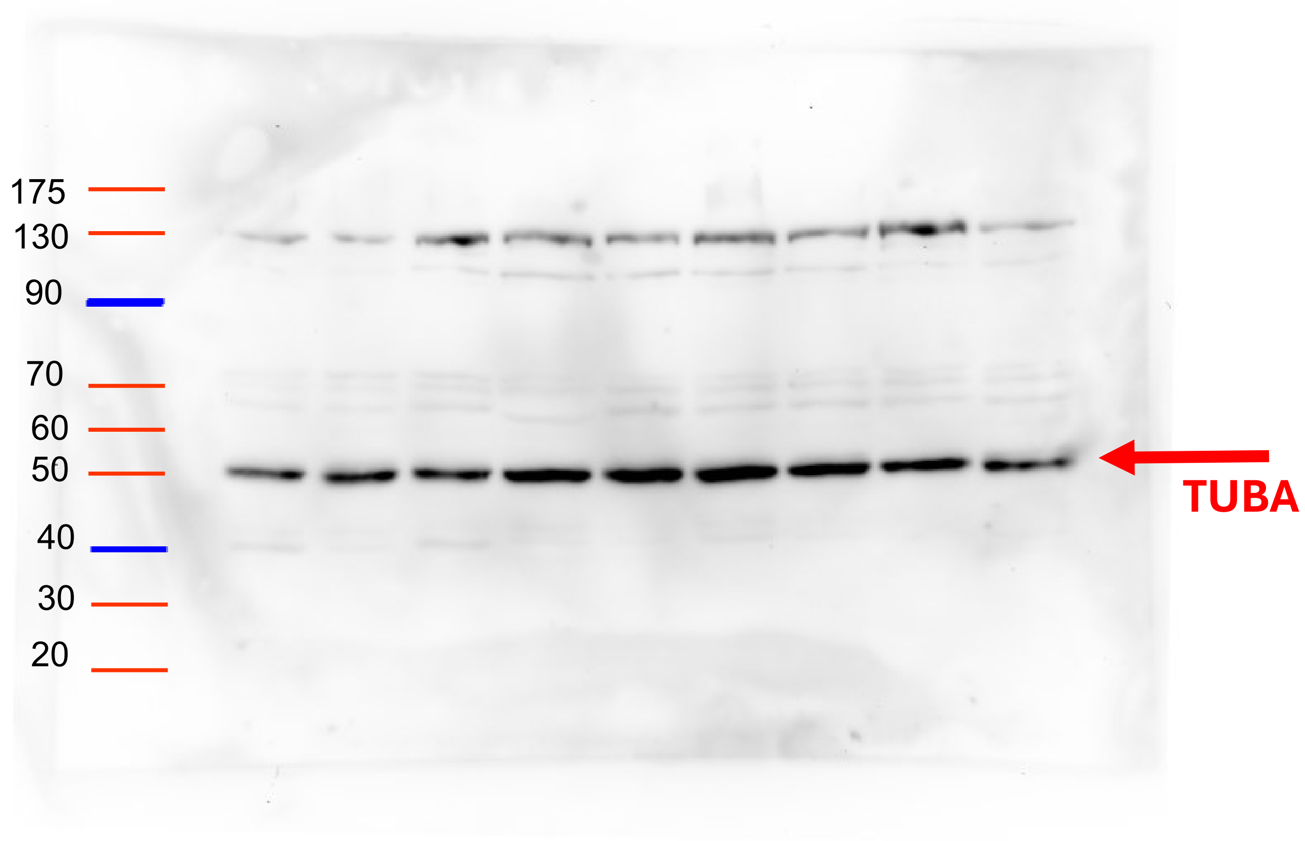

SDS-PAGE GEL 8%

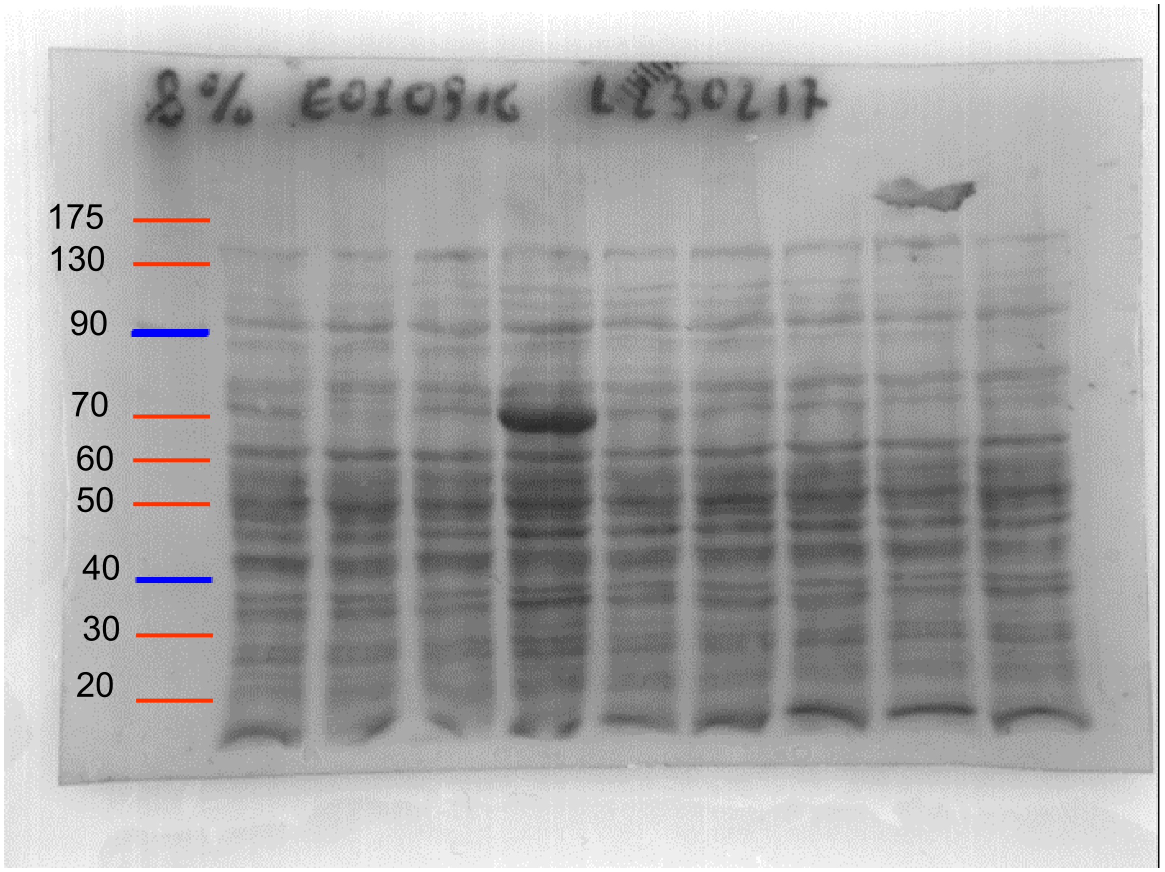

Amid Black

TUBA FIGURE 8C
PANEL 4

SECOND ADRP BLOT

OVEREXPOSURE

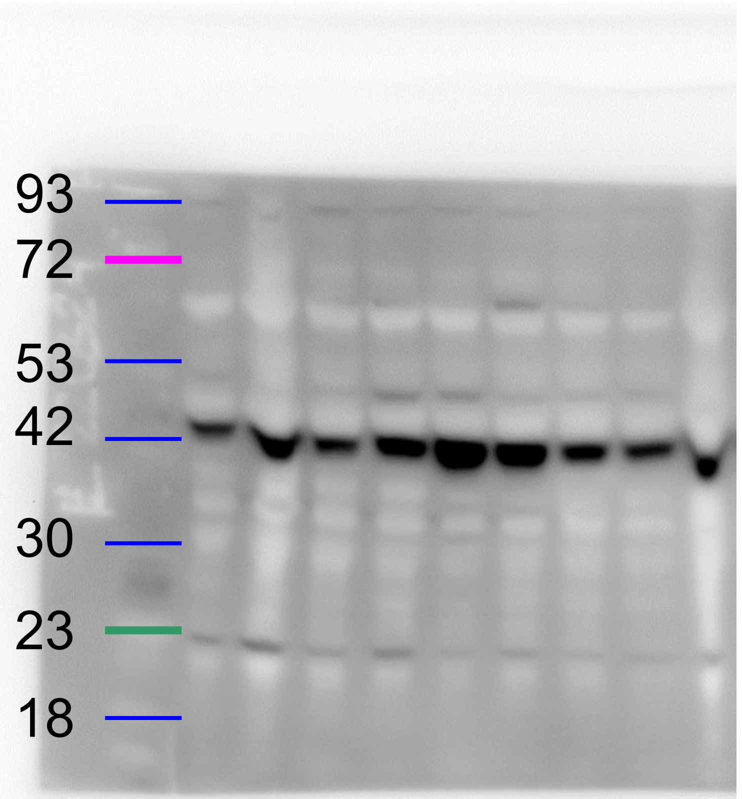

NuPAGE Bis-Tris GEL 4-12%, lower part

EXPOSURE: 60s

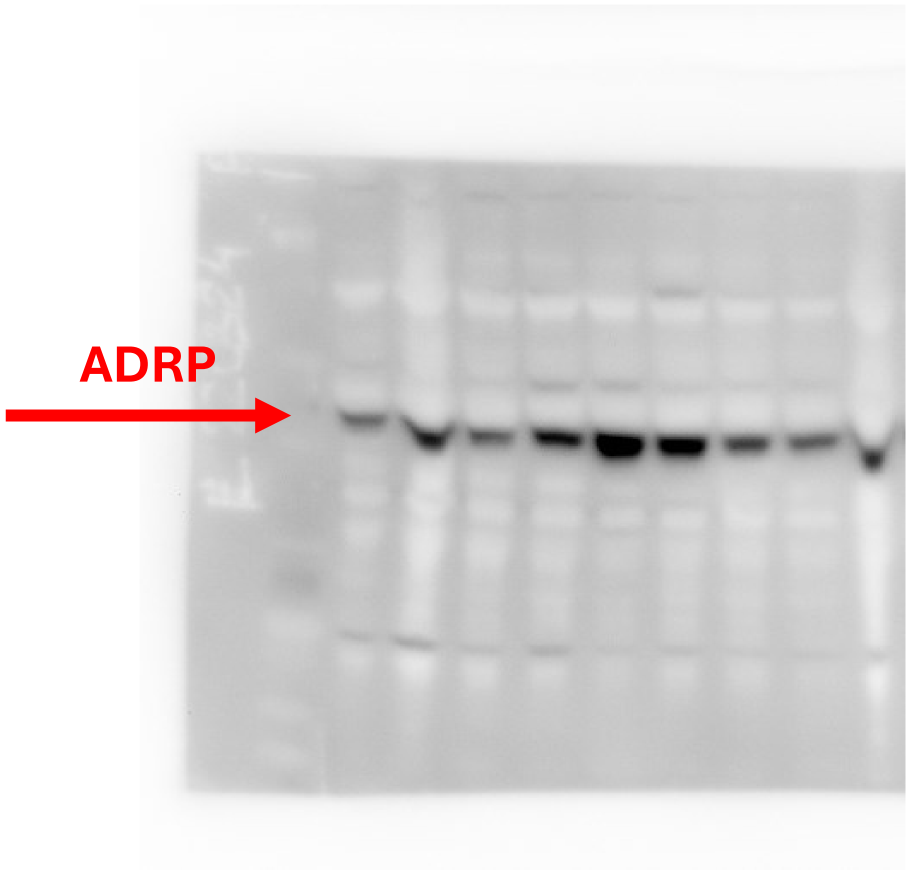

EXPOSURE: 60s

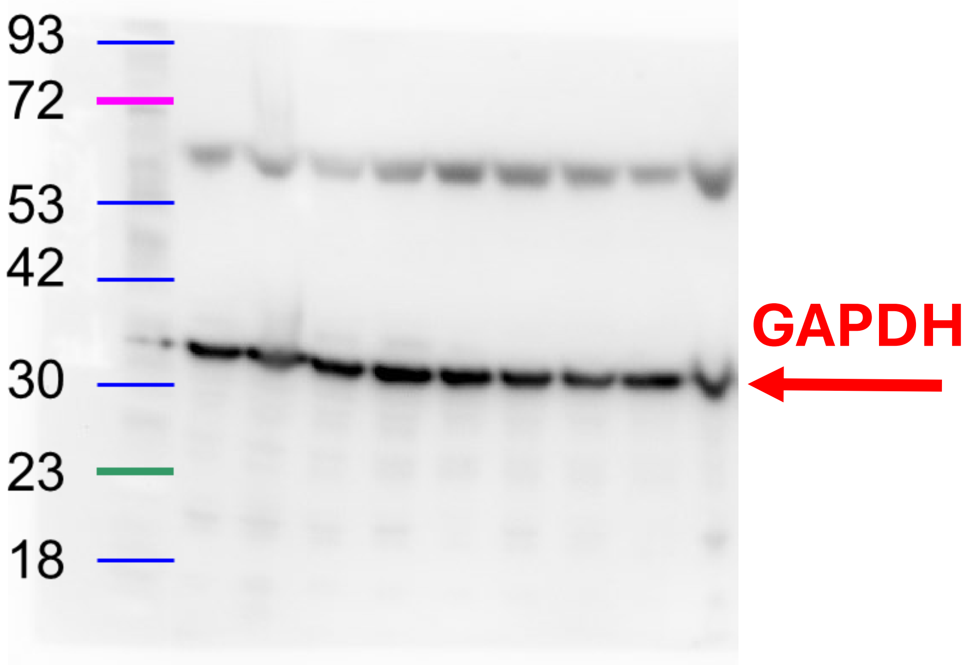

Amid Black

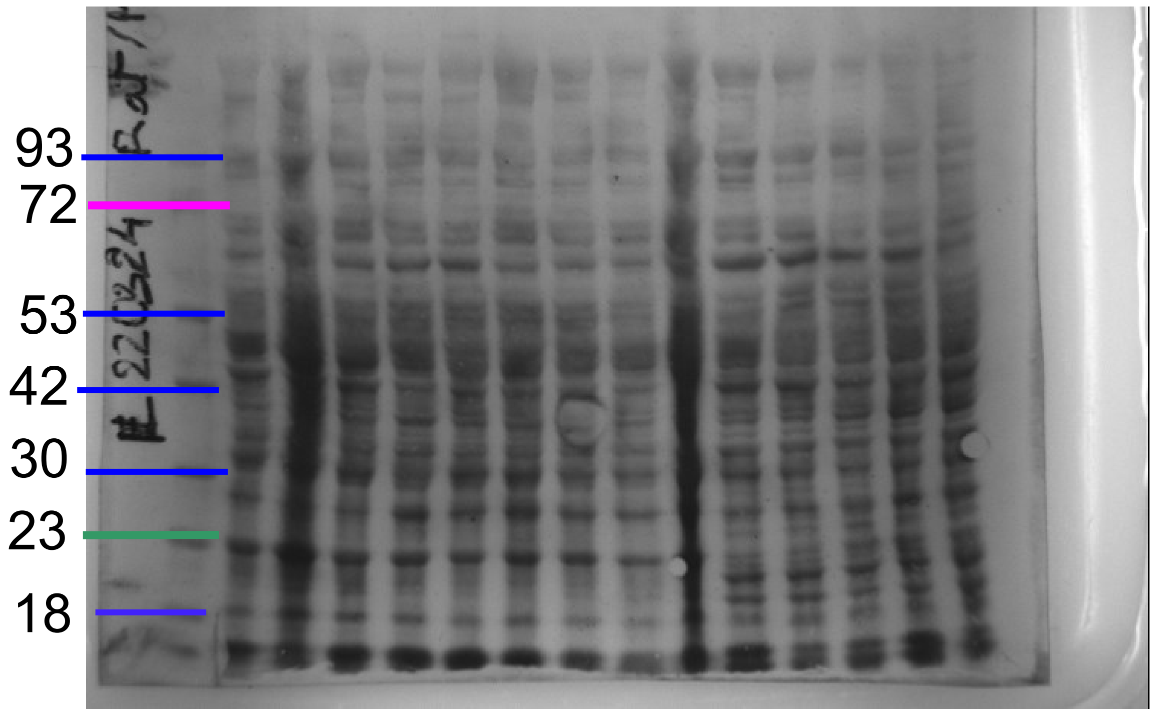

Supplement: Supplementary file 1 [file antioxidants-13-00766-s001.zip › antioxidants-2976075-supplementary.pdf]
